# *Dolichoperoides macalpini *(Nicoll, 1914) (Digenea: Dolichoperoididae) infecting venomous snakes (Elapidae) across Australia: molecular characterisation and infection parameters

**DOI:** 10.1007/s00436-022-07502-x

**Published:** 2022-04-01

**Authors:** Diane P. Barton, Damian C. Lettoof, Simon Fearn, Xiaocheng Zhu, Nidhish Francis, Shokoofeh Shamsi

**Affiliations:** 1 School of Agricultural, Environmental and Veterinary Sciences, Charles Sturt University, Wagga Wagga, New South Wales Australia; 2 School of Molecular and Life Sciences, Curtin University, Perth, Western Australia Australia; 3 Natural Sciences, Queen Victoria Museum and Art Gallery, Launceston, Tasmania Australia; 4 New South Wales Department of Primary Industries, Wagga Wagga Agricultural Institute, Wagga Wagga, New South Wales Australia

**Keywords:** Host-parasite dynamics, Dolichoperoididae, Australia, Reptile, Biodiversity

## Abstract

Specimens of *Dolichoperoides macalpini* (Nicoll, 1914) (Digenea: Dolichoperoididae) were collected from Australian venomous snakes (Elapidae): *Notechis scutatus* Peters, 1861 and *Austrelaps superbus* (Günther, 1858) from Tasmania and surrounding islands and *N. s. occidentalis* Glauert, 1948 from wetlands near Perth, Western Australia. Despite variation in morphological measurements, genetic analysis showed that the one species of digeneans infected the snakes from all locations. This study presents the first DNA sequences for *D. macalpini* (internal transcribed spacer, 18S, 28S), confirming its placement in a family separate from the Reniferidae and Telorchiidae. Analysis of the infection dynamics of infection in Western Australian snakes showed significant differences in levels of infection between wetland locations, season and year of collection. Infection of *D. macalpini* was reported in the gastrointestinal tract, including the mouth, in freshly euthanised snakes in Western Australia, and in the lung in Tasmanian snakes, consistent with earlier reports. Differences in morphology and site of infection are suggested to be due to a combination of season and maturity of the digenean, with infection potentially occurring early in the season, as the snakes emerge from torpor. The need for research on the seasonal dynamics of infection with this parasite is discussed.

## Introduction

Elapid snakes are a well-known component of the Australian reptile fauna (Shine 1991). However, their parasite fauna is not as well known. Of the species currently recorded parasitising terrestrial elapids — in Pichelin et al. (1999) — the only reported digenean parasite is *Dolichoperoides macalpini* (Nicoll 1918).

Specimens of *D. macalpini* were first reported in the trachea and gullet of a copperhead snake (now identified as *Austrelaps superbus* (Günther, 1858)) collected in Victoria by McAlpine (1891). Despite McAlpine (1891) providing a detailed morphological description, the collected specimens were not identified until the study of Nicoll (1918). Examination of the original specimens, supplemented by new collections from an unidentified snake (subsequently reported as *Notechis scutatus* Peters, 1861 in Johnston (1918)) from Tasmania, led to the description of *Dolichopera macalpini* Nicoll 1918, which was similar in morphology to *Dolichopera parvula* Nicoll 1914, described from the oesophagus of a python in Queensland (Nicoll 1914). Johnston and Angel (1940) subsequently determined that the differences in morphology were sufficient to erect a new genus, *Dolichoperoides* Johnston and Angel 1940, for the specimens collected from the elapid snakes. Within *D. macalpini*, Johnston and Angel (1940) included digeneans collected from several snake species and localities (Johnston 1910, 1911; Johnston and Cleland 1937; Johnston and Angel 1940).

Examination of a collection of snakes from Tasmania provided additional specimens of digeneans corresponding to *D. macalpini*. Concurrently, a separate project examining tiger snakes around Perth, Western Australia, also provided specimens corresponding to *D. macalpini*. In this study, we examine the Western Australian specimens in conjunction with the Tasmanian specimens to confirm their identification through both morphological and molecular characterisation, as. *D. macalpini* has not previously been reported from Western Australia.

## Materials and methods

### Host collection

**Fig. 1:**
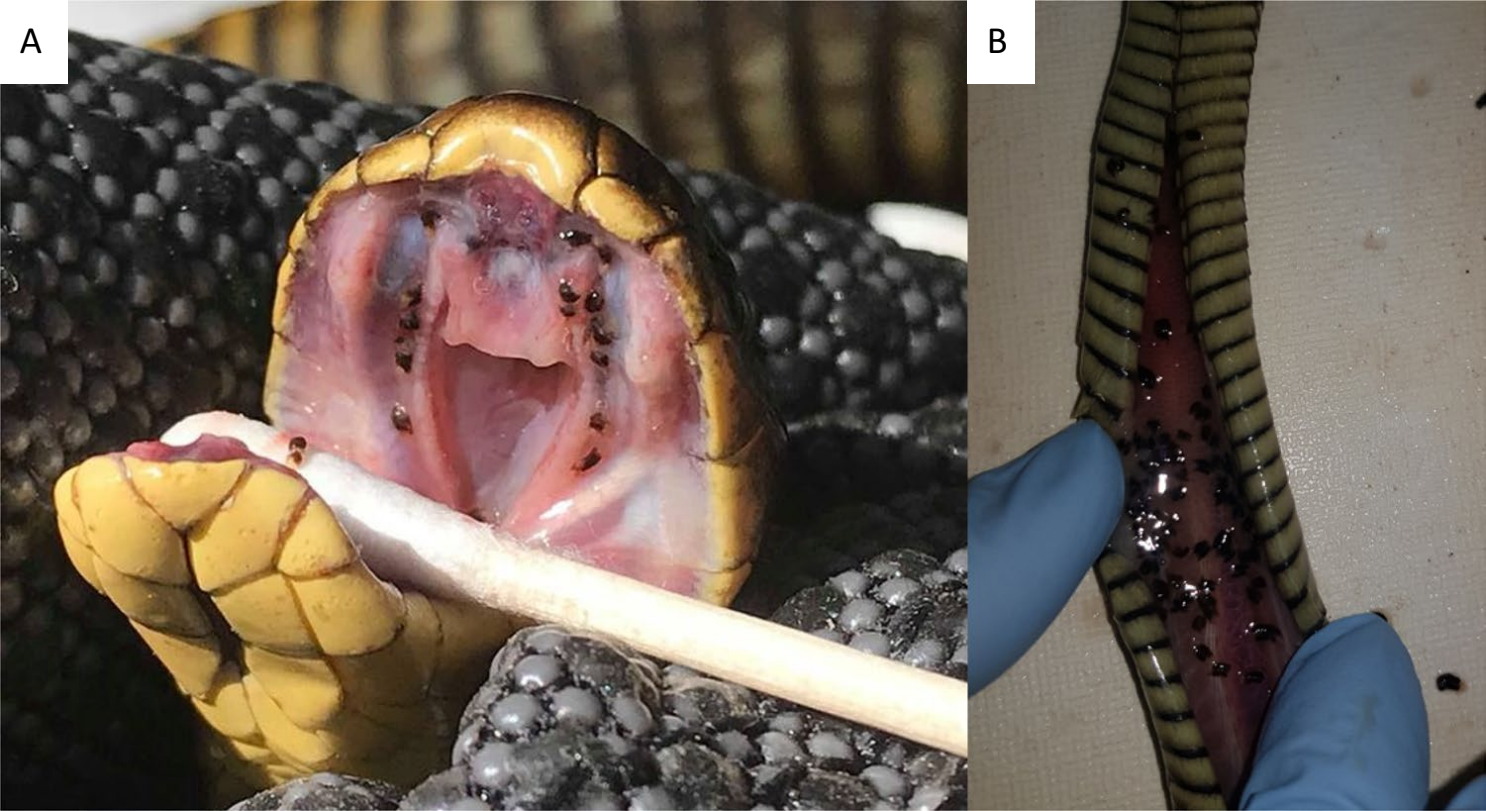
Specimens of *Dolichoperoides macalpini* in the mouth of a live *Notechis scutatus occidentalis* collected in Perth, Western Australia (**A**) and in the lungs of a *Notechis scutatus* collected from near Lulworth, northern Tasmania (**B**)

A total of 38 snakes were collected from locations across Tasmania and the Bass Strait Islands between January 2012 and December 2018: *Notechis scutatus* (*N* = 21), *Austrelaps superbus* (*N* = 16) and *Drysdalia coronoides* (Günther, 1858) (*N* = 1) (Table 1). Most snakes were collected as road-killed specimens, but three *A. superbus* were collected live and kept in captivity until deceased; all snakes were frozen at time of collection/death. The snout-vent length (*SVL*; mm), tail length (*TL*; mm) and weight (g) were measured and the sex determined at the time of collection/death. All snakes were in the collection of the Queen Victoria Museum and Art Gallery, Launceston, Tasmania, in April 2019. At the time of dissection, the mouth was examined for parasites (Fig. 1A) prior to the removal of all internal organs. The lung and trachea were opened longitudinally and examined (Fig. 1B). The gastrointestinal tract, from the beginning of the oesophagus to the rectum, was opened longitudinally and examined. All digeneans were collected in 70% ethanol.

**Table 1:** Information on the snakes examined in this study. Data is presented as mean with range in parentheses except for infection data. *F* female, *Juv* juvenile, *M* male, *N* number of snakes, *NP* National Park, *P* prevalence of infection (%), *SVL* snout-vent length (mm), *TL* tail length (mm), *WA* Western Australia, *Wt* weight (g)

Snake species	Location	Sex	*N*	*SVL*	*TL*	*Wt*	No. infected (*P*)	Intensity
*Notechis scutatus occidentalis*	Bibra Lake, WA	M	10	807.3 (746–847)	96.7 (68–137)	258.1 (127–328.8)	2 (20%)	3.5 (2–5)
		F	6	695.8 (580–837)	103.5 (61–140)	199.8 (105–305)	1 (16.7%)	2
	Herdsman Lake, WA	M	14	814.1 (671–933)	86 (35–118)	215.3 (125–338.5)	5 (35.7%)	45.6 (27–108)
		F	7	176.2 (698–790)	92.7 (68–117)	176.2 (117–261.8)	3 (42.9%)	36.3 (4–109)
	Lake Joondalup, WA	M	9	783.8 (724–847)	106.9 (58–142)	237.9 (165–327)	9 (100%)	52.3 (14–89)
		F	6	684.3 (642–712)	85.5 (28–111)	166.4 (110–220)	6 (100%)	49.5 (22–84)
	Yanchep NP, WA	M	6	795.2 (670–899)	105.7 (66–142)	232.3 (140–369.5)	6 (100%)	64 (18–130)
		F	4	699.3 (650–766)	99 (67–114)	160.6 (114–208.3)	4 (100%)	71.8 (26–144)
*Notechis scutatus*	Tasmania	M	6	1070.2 (895–1416)	186.3 (145–220)	749.4 (375.1–1400)		
		F	15	992.8 (415–1375)	172.7 (80–235)	760.2 (33–2450)	1 (6.7%)	64
*Austrelaps superbus*	Tasmania	M	7	763.9 (527–885)	155.1 (127–190)	380.3 (88.5–700)	2 (28.6%)	1.5 (2–3)
		F	6	684.2 (400–845)	138.8 (80–165)	225.9 (39.6–371.2)	1 (16.7%)	25
		Juv	3	308.3 (160–435)	56.3 (37.5–75)	84.3 (3.5–209)		
*Drysdalia coronoides*	Tasmania	F	1	415	102.5	83

A total of 62 western tiger snakes (*N. s. occidentalis*) were collected and euthanised from four wetlands in and surrounding Perth, Western Australia: Bibra Lake (*N* = 16), Herdsman Lake (*N* = 21), Lake Joondalup (*N* = 15) and Loch McNess within Yanchep National Park (*N* = 10). Snakes were collected in 2018 (*N* = 3), 2019 (*N* = 24) and 2020 (*N* = 35) (Table 1). Collections occurred over a 4-week period from March to April in 2019 (autumn; *N* = 21), and a 6-week period from September to October (spring; *N* = 41) each year. All sites were located within a 60-km north-to-south range; detailed descriptions of the collection sites are available in Lettoof et al. (2020a). Snakes were collected by hand; *SVL*, *TL* and weight were measured and the sex identified via probing of the hemi-penal pocket (males; *N* = 39, females; *N* = 23). Parasites were examined and counted using the same method as above.

### Parasite identification

Morphological identification: Specimens were stained in acetocarmine, dehydrated through a graded ethanol series, cleared in xylene and mounted in Canada balsam. Measurements were taken from a compound microscope with an eyepiece micrometer. Measurements are presented as the mean with the range in parentheses and are in micrometres, unless otherwise stated. Specimens were identified using the descriptions and keys available in the literature (Crowcroft 1949; Gibson 2008; Nicoll 1918).

Whole-mount specimens have been deposited in the QVMAG (QVM 2019:19:0001–0004) and Western Australian Museum (WAM V10996-V10998).

#### Genetic characterisation

The posterior extremity was cut from representative specimens collected from *N. scutatus occidentalis* in Western Australia, *N. scutatus* in Tasmania and *A. superbus* in Tasmania for molecular analysis. Genomic DNA was extracted using the Qiagen DNeasy kit according to the manufacturer’s instructions. The 18S rRNA sequences were amplified and sequenced using two primer combinations (primer sets WormA and 1270R and 1100F and WormB) (Littlewood and Olson 2001). Another two primer pairs, LSU-5 m and ECD2m and 300Fm and 1500Rm (Olson et al. 2003), were used to obtain 28S rRNA sequences. The internal transcribed spacer (ITS) sequences were obtained using a pair of primers designed in this study (18SDigenea-F1 5′-GTCGTAACAAGGTTTCCGTAGG-3′ and 28SDigenea-R1 5′-GTGATATGCTTAAGTTCAGCGG-3′). The chromatogram of all sequences was quality checked and assembled using SeqMan Pro ver. 8.1 (DNASTAR Inc.). Our sequences were deposited in GenBank under accession numbers OK572359–OK572368 (18S and 28S) and OM568836–OM568839 (ITS).

Sequences of closely related species of the superfamily Plagiorchioidea were obtained from GenBank, using BLAST results, for phylogenetic analyses (Table 2). Species of *Macvicaria* Gibson and Bray, 1982 from the same suborder Xiphidiata, but the superfamily Opecoeloidea, were used as an outgroup in the phylogenetic analyses. The GTR + I + G model was selected as the best-fit evolutionary model for 18S and 28S regions, and the GTR + G model was used for the ITS region as inferred by jModelTest 2 (Darriba et al. 2012). The phylogeny of selected sequences was inferred using MrBayes 3.2 for 2,000,000 generations until the average standard deviation is lower than 0.005. The tree was visualised using FigTree v 1.4.3 (Rambaut 2014).

**Table 2:** Details of the sequences generated in this study

Species	Host species	Accession number			Locality
		18S	28S	ITS	
*Dolichoperoides macalpini*	*Austrelaps superbus*	OK572359–60	OK572367–68	OM568836	Tasmania, Australia
	*Notechis scutatus*	OK572362–63	OK572365–66	OM568838–39	Tasmania, Australia
	*Notechis scutatus occidentalis*	OK572361	OK572364	OM568837	Perth, Western Australia

### Infection data analysis

As the western tiger snakes were collected from four isolated wetlands, we assessed patterns of infection between sites. First, we determined if there was a difference in infection intensity among sites and years, and between seasons. We ran a generalised linear model (GLM; Poisson error structure, square-root link) with total count (mouth and oesophagus/stomach combined) as the response variable, and site, year and season as the predictor variables. Total counts were not significantly different between host sexes (*X*^2^ = 2.57, *p* = 0.11), so data were pooled. We then compared the relationship between western tiger snake digenean counts in the mouth and oesophagus/stomach, using a generalised linear mixed model (GLMM; Poisson error structure, square-root link) with counts in the mouth as the response variable, counts in the oesophagus/stomach as the predictor variable and both site and season as random effects. Finally, we compared the relationship between snake *SVL* (a proxy for age) and total infection using a GLMM (Poisson error structure, square-root link) with total count as the response variable, *SVL* as the predictor variable and site as a random effect. Analyses were conducted in version 4.0.3 using the lme4 package (Bates et al. 2014).

Due to the low number of infected snakes, and inconsistencies in the collection of data, the Tasmanian snakes were not included in any statistical analyses.

## Results

### Parasite identification

Specimens were identified as *D. macalpini* based on the host species, location of infection (mouth, oesophagus, stomach, intestine and lung) and comparison with the descriptions of Nicoll (1918) and Crowcroft (1949) (Table 3) and the key provided by Gibson (2008). The position of the ventral sucker close to the middle of the body, the testes in the posterior half of the hindbody, the large cirrus sac and the extent of the uterine coils into the hindbody confirmed the identification as *D. macalpini*.

**Table 3:** Measurements of specimens of *Dolichoperoides macalpini* collected from this study^a^ in comparison to measurements presented in the literature

Reference	This study	Nicoll (1918)	Johnston and Angel (1940)	Crowcroft (1949)
Host species	*Notechis scutatus occidentalis*	*Notechis scutatus* *Austrelaps superbus*	*Notechis scutatus* *Austrelaps superbus*	*Notechis scutatus*
Location	Western Australia	Victoria & Tasmania	New South Wales, Victoria, South Australia & Tasmania	Tasmania
Body length (mm)	2.0 (1.6–2.4)	3.1 (2.6–3.5)	2.0–4.8	Up to 4.0
Body width (mm)	0.61 (0.6–0.7)	1.25 (1.0–1.6)	1.3–1.7	Up to 1.6
Oral sucker diameter	326.7 (290–380)	315–465	300–450	600 × 490
Ventral sucker diameter	290 (270–320)	250–450	250–450	470
Pharynx diameter	136.7 (120–150) × 123.3 (110–150)	150 × 135	140	180
Testes length	238.3 (200–280)	230	330–400	450
Testes width	130 (110–150)	135	150–180	230
Cirrus sac length			1300	1000
Cirrus sac width			200–250	300
Ovary diameter	80	75	140–200	250
Egg length	34.2 (31.25–36.25)	28–32	28–34	36
Egg width	17.5 (16.25–18.75)	18–19	18–19	20

^a^Specimens collected from Tasmanian snakes were not included in the table as poor quality of the specimens prevented measurements of internal structures

Comparison of measurements of specimens collected in this study showed variability. The specimens collected from Western Australian snakes were considerably smaller (body length 1.6–2.4 mm) than those collected from Tasmanian snakes (3.2–4.1 mm). However, all specimens contained a developed uterus with eggs, although the extent of the distribution of the uterus in the Tasmanian specimens was much greater, obscuring most of the internal organs.

**Fig. 2:**
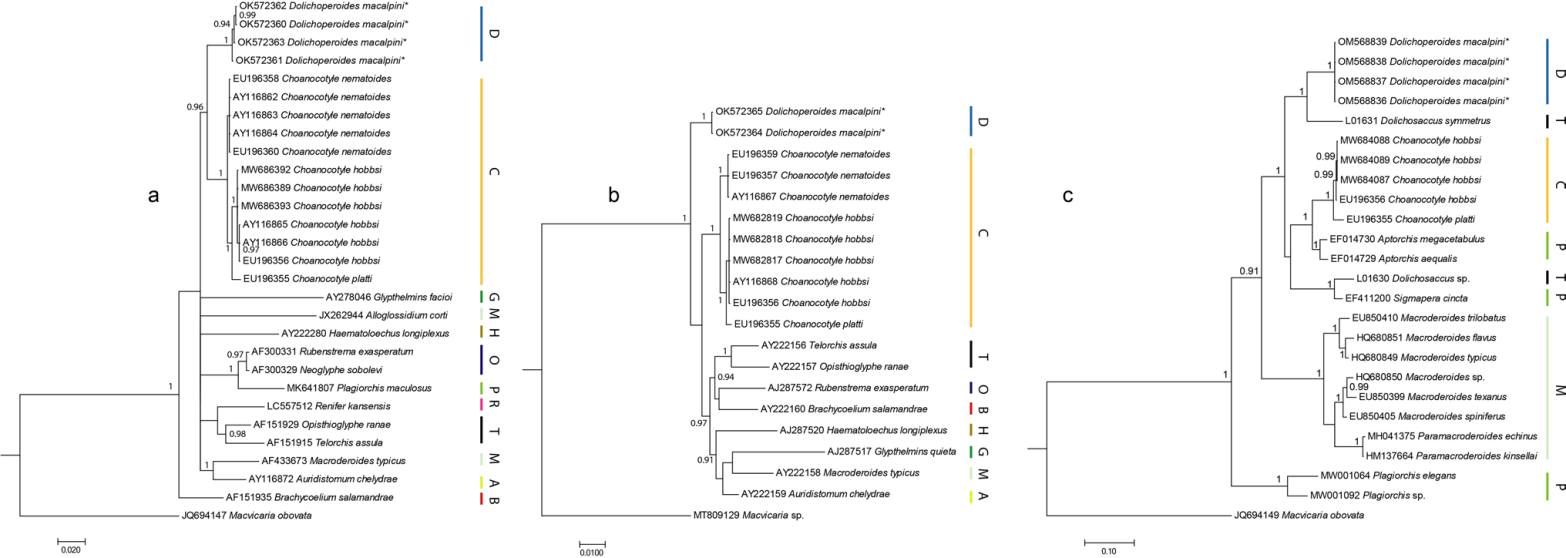
Phylogenetic placement of *Dolichoperoides macalpini* among closely related species from superfamily Plagiorchioidea based on partial 28S (**A**), 18S rRNA (**B**) and ITS (**C**) sequences. Trees were calculated through Bayesian algorithms. Posterior probabilities (Bayesian tree) over 0.90 are shown on the node. Sequences generated in this study are denoted by an asterisk. Families to which genera belong are indicated by colour-coded lines; the colour codes are the same across all three phylogenetic trees. A Auridistomidae, B Brachycoeliidae, C Choanocotylidae, D Dolichoperoididae, G Glypthelminthidae, H Haematoloechidae, M Macroderoididae, O Omphalometridae, P Plagiorchiidae, R Reniferidae, T Telorchiidae

DNA sequences of the 18S and 28S rRNA regions were generated from five *D. macalpini* specimens; ITS sequences were successfully generated from four of these specimens. Only obtained sequences that covered the full sequence region were used in the phylogenetic analyses. Shorter sequences that were obtained from this study were identical to the longer ones but were too short to be included in the analyses. The 18S rRNA and ITS sequences were identical among specimens, but there were slight differences (1–2-bp differences out of 1331 bp) among the 28S rRNA sequences. These specimens formed a distinct group in all of the phylogenetic analyses (Fig. 2) relative to species of the Plagiorchioidea. Within the ITS phylogenetic tree (Fig. 2C), however, the sequences for *D. macalpini* grouped with a sequence for *Dolichosaccus symmetrus* (Johnston, 1912) (L01631), collected from a toad, *Rhinella marina* (Linnaeus, 1758), in northern Queensland (Luton et al. 1992). This clade was then separated from the other species of the Plagiorchioidea, including *Dolichosaccus* sp. (L01630).

### Infection data

#### Tasmanian snakes

Digeneans were detected in the lung and/or mouth of all infected snakes, with one *A. superbus* also having a few digeneans present in the intestine. Four of the 38 (10.5%) snakes were infected with *D. macalpini* (Table 1). *Austrelaps superbus* were more often infected, with 3 of the 16 (14.3%) snakes infected. All three infected snakes were collected from an 80-km stretch of the Tasman Highway along the eastern coast of Tasmania, south of the township of Bicheno. Two male *A. superbus* and a female were infected. The single infected female *N. scutatus* (4.8% of 21 N*. scutatus* collected) was collected from north-eastern Tasmania, near the township of Lulworth.

#### Western Australian tiger snakes

**Fig. 3:**
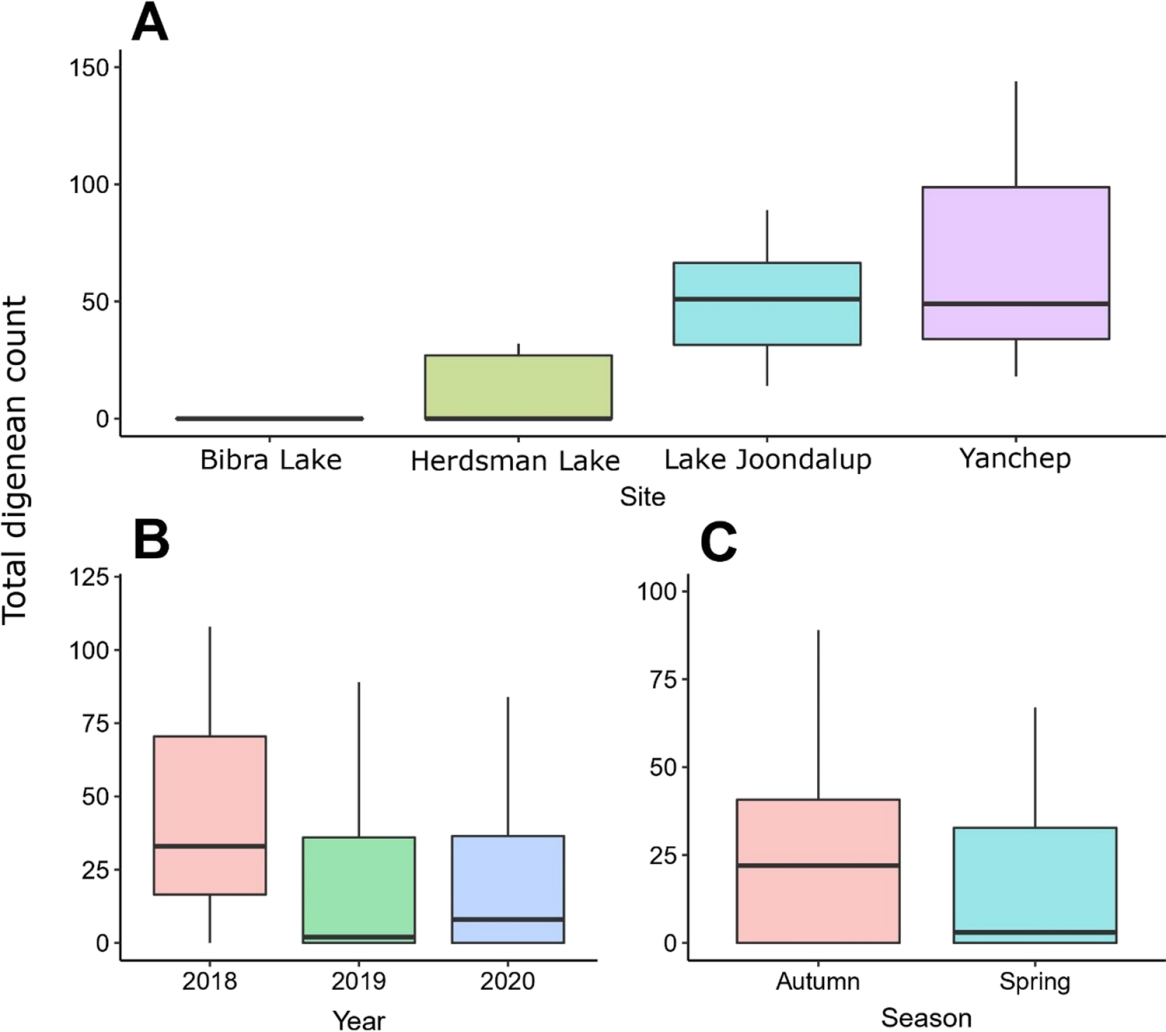
Total infection of *Dolichoperoides macalpini* in 62 *Notechis scutatus occidentalis* collected from four sites around Perth, Western Australia; categorised by site (**A**), year (**B**) and season (**C**)

Digeneans were only detected in the mouth and oesophagus/stomach, and no digeneans were detected in the lungs or intestinal tract of *N. s. occidentalis*. Prevalence ranged from 16.4 to 100%, depending on the wetland site at which the snakes were collected. Total digenean infection was significantly different among sites (*r*^2^ = 0.97, *X*^2^ = 1505.28, *p* < 0.01) and years (*r*^2^ = 0.97, *X*^2^ = 145.65, *p* < 0.01) and between seasons (*r*^2^ = 0.97, *X*^2^ = 73.53, *p* < 0.01). Bibra Lake snakes had the lowest intensity, followed by Herdsman Lake, Lake Joondalup and Yanchep; intensity was higher in autumn than in spring, and 2019 had lower infection than 2018 and 2020 (Fig. 3). There was a significant positive relationship between mouth infection and oesophagus infection (*r*^2^ = 0.89, *X*^2^ = 12.86, *p* < 0.01). *SVL* was not a significant predictor of total digenean intensity (*r*^2^ = 0.97, *X*^2^ = 1.94, *p* < 0.16).

## Discussion

This study has provided the first molecular characterisation of *D. macalpini*. Previous authors (see Crowcroft (1949)) have suggested that the morphological variation between specimens collected in Tasmania and those from mainland Australia might indicate separate species. Similar morphological variation was found in the overall body measurements for specimens collected from Tasmanian snakes and those from Western Australia in this study. However, the ITS sequences for specimens collected from both locations showed 100% similarity. Thus, the one species, *D. macalpini*, infects a range of elapid snakes across the Australian continent. No genetic sequences for *D. parvula*, the other species within the Dolichoperoididae, were available for comparison.

The placement of the specimens in the trees generated in this study supports the placement of *Dolichoperoides* within its own family and separate from representatives of the families Reniferidae and Telorchiidae, as proposed by Johnston and Angel (1940). In the 28S rRNA tree, the genus appeared as a sister group to the Choanocotylidae, which are parasitic in Australian freshwater turtles (Tkach 2008), but, surprisingly, in the 18S rRNA tree, it appeared as the basal group to the rest of the plagiorchoideans. The patterns of distribution of species in the 18S rRNA tree was different from that in the 28S rRNA tree in many respects, with *Brachycoelium salamandrae* (Frölich, 1789) appearing as a sister taxon to *Rubenstrema exasperatum* (Rudolphi, 1819) in the 18S rRNA tree, but was the basal group in the 28S rRNA tree. Despite these variations, the overall patterns mirror the results of Olson et al. (2003), with the sequences forming a monophyletic clade.

In the ITS tree, however, *D. macalpini* grouped with a sequence of *Do. symmetrus*, a member of the Telorchiidae. This group was basal to a mixed group of Choanocotylidae, Plagiorchiidae and Telorchiidae, all of which were collected from Australian amphibians and reptiles. Barton (1994) found that the *Do. symmetrus* collected from toads in Australia had slight, but consistent, morphological variation from the original description and were morphologically quite distinct from other species of *Dolichosaccus* Johnston, 1912 in Australia. Luton et al. (1992), also noted  that the sequence of *Do. symmetrus* was very different from the sequence of *Dolichosaccus* sp. (also from the toad in Australia and subsequently described as *D. helocirrus* by Barton (1994)) which was found in the mixed group of the tree in this study. Thus, the *Do. symmetrus* collected from toads in Australia may be a misidentification and requires re-examination, along with other specimens of *Dolichosaccus* collected from other hosts, with molecular sequences.

In the 28S rRNA and ITS trees generated in this study, digeneans collected from Australian hosts grouped together into a clade separate from species collected from elsewhere in the world. Further work is needed to sequence a larger variety of digeneans from Australian reptiles and amphibians to determine if this relationship holds true.

This study also provides preliminary infection data, for this parasite. The intensity of infection in the Tasmanian snakes was relatively low, compared to that found in the Western Australian snakes. This may be due to the Tasmanian snakes being collected as road-killed specimens, with an unknown time between death and collection. As such, parasites may have already exited from the host — as described by McAlpine (1891) — affecting the parasite infection levels. The Western Australian snakes, on the other hand, were examined shortly after euthanasia and had much heavier infections. Despite their heavy infections, there was no relationship between infection level and body condition for the Western Australian snakes (Lettoof et al. 2022).

Further research is needed to determine the potential biological and/or ecological factors contributing to infection with *D. macalpini*. As this parasite is transmitted via an encysted metacercarial stage found in frogs (Johnston and Angel 1940), it is of interest to note differences in diets between snakes in the Western Australian and Tasmanian locations. Snakes in Western Australia were found to prey predominately on frogs (almost 90% of prey items recovered) (Lettoof et al. 2020b), whereas the stomach contents of the Tasmanian tiger snakes included rodents and birds, and the *A. superbus* included small lizards with no frog remains noted (Barton, pers. obs.). Tasmanian *N. scutatus* specialise on endothermic prey (mainly mammals and birds) after adulthood, and this appears to be related to the high densities of small mammals in many Tasmanian habitats. In contrast, Tasmanian *A. superbus* specialise on large volumes of small ectothermic prey (frogs and reptiles) in all habitats (Fearn et al. 2012). The trophic ecology of Tasmanian *A. superbus* is therefore more similar to Western Australian *N. s. occidentalis* than sympatric populations of Tasmanian *N. scutatus*, which may explain the differences in infection between the Tasmanian snake species, with *A. superbus* being more heavily infected.

The analysis of the Western Australian snakes found a difference in infection levels between spring (September to November) and autumn (March to May). In southern Australia, elapid snakes are generally active between September and May and quiescent between June and August (Greer 1997). Johnston and Angel (1940) found infections of *D. macalpini* in the intestine of *N. scutatus* in South Australia to occur from August to October and February to May, suggesting infection could occur at any time over the active “season”. In their experimental infections, they found that snails only became infected in September, suggesting that spring was the “normal time” for snails to be infected (Johnston and Angel 1940). This may account for the lower levels of infection in Western Australian tiger snakes in spring. Differences in infection levels were also related to the location of collection in Western Australia, with the more heavily urbanised locations of Bibra and Herdsman Lakes (Lettoof et al. 2022) having significantly lower levels of infection. Given the reliance on aquatic intermediate hosts (Johnston and Angel 1940), and the potential effect of urbanisation and pollution on the survival of frogs (the second intermediate host in the life cycle), reduction in infection of *D. macalpini* in tiger snakes may be a reflection of environmental impacts on the populations of intermediate hosts.

Throughout its history, *D. macalpini* has been reported from various locations in the body, from the intestine to the lung, with Nicoll (1918) commenting on the unusualness of finding a species of parasite to occupy both locations. Interestingly, *D. macalpini* was never collected from the lungs of the Western Australian snakes, in contrast to most of the Tasmanian specimens. The reasons behind this are not known, although we can offer some speculation. It is possible that the parasites move location after the death of the host, as suggested by McAlpine (1891) where the digeneans were “…evidently making their way out of the body”. The Tasmanian snakes in this study, however, were collected post-death, whereas the Western Australian snakes were freshly euthanised, and digeneans were also observed in the mouths of live snakes (DC Lettoof, pers. obs.). Therefore, post-death migration is unlikely as an explanation. Alternatively, these different locations within the host may relate to the life cycle and/or seasonal dynamics of infection of *D. macalpini* in snakes and may also help to explain the variation in morphological dimensions between specimens collected from Western Australian and Tasmanian snakes. The specimens collected from Western Australia examined in this study were collected early in the season, and the infected *N. scutatus* collected in Tasmania was collected in February, i.e. later in the season. Johnston and Angel (1940) noted that only specimens larger than 500 μm were found in the lung and oesophagus, suggesting that more mature specimens are located in these sites. How long the digeneans may survive is unknown. More research is required to document the infection levels of these parasites in a population of snakes over a year to determine if there is a seasonal pattern to movement within the snakes.

As mentioned above, more research is required to establish and understand the patterns of parasite infections in snakes, considering the different biology and ecology that can occur between geographical locations and species. Additionally, further samples of digeneans collected from other elapid snakes in different regions of Australia need to be molecularly characterised to determine the full distribution of *D. macalpini*.

## Data Availability

Specimens have been deposited in recognised curated museum collections. Genetic sequences have been deposited in GenBank.
